# Long‐Term Determinants of Lean Mass in Ageing Adults: A 17‐Year Longitudinal Cohort Study With Repeated Measures

**DOI:** 10.1111/ajag.70242

**Published:** 2026-10-12

**Authors:** Aminu Suleiman, Mojtaba Lotfaliany, Lana J. Williams, Kara B. Anderson, Emma C. West, Amanda L. Stuart, Julie A. Pasco

**Affiliations:** ^1^ Deakin IMPACT – The Institute for Mental and Physical Health and Clinical Translation, School of Medicine Deakin University Geelong Victoria Australia; ^2^ Barwon Health Geelong Victoria Australia; ^3^ School of Public Health and Preventive Medicine Monash University Melbourne Victoria Australia

**Keywords:** body composition, cohort study, healthy aging, lifestyle, linear model

## Abstract

**Objective:**

Age‐related loss of lean mass is a hallmark of sarcopenia and a contributor to functional decline and morbidity in ageing populations. This study aimed to identify long‐term determinants of lean mass during ageing.

**Methods:**

Data from 3033 participants (ages 20–96 years; 49% women) in the Geelong Osteoporosis Study were analysed using linear mixed effect models with random intercepts. Appendicular lean mass (ALM) was derived from arm and leg lean mass measured using dual‐energy X‐ray absorptiometry. Time‐varying covariates were included; missing data were imputed using predictive mean matching.

**Results:**

Over 16.7 years (IQR 9.7–23.2), women and older participants had lower ALM (*β* = −8.36; 95% CI –8.58, –8.15; *β* = −0.07; 95% CI –0.08, –0.06). Higher ALM was associated with socio‐economic status (0.33; 0.14, 0.51), dietary calcium intake (0.46; 0.20, 0.67), femoral neck BMD (0.38; 0.29, 0.47) and thyroid hormone therapy (0.52; 0.04, 0.99). Physical inactivity (−0.48; −0.65, –0.31), smoking (−0.24; −0.46, –0.06) and stroke history (−0.51; −0.96, –0.07) were associated with lower ALM. Non‐linear associations were observed for follow‐up duration (fu: *β* = −0.16; fu^2^: *β* = 0.01; −*p* < 0.001), body mass index (BMI: *β* = 0.55; BMI^2^: *β* = −0.00; *p* < 0.001), systolic blood pressure (SBP: *β* = 0.06; SBP^2^: *β* = −0.00; *p* < 0.001), serum C‐terminal telopeptide of type I collagen (CTx: *β* = 0.14, *p* < 0.001; CTx^2^: *β* = −0.01, *p* = 0.03) and high‐sensitivity Creactive protein (hsCRP: *β* = −10.38; hsCRP^2^: *β* = 11.70; −*p* < 0.001). In fat‐mass‐adjusted models, low‐density lipoprotein cholesterol (0.14; 0.04, 0.24) and triglycerides (0.22; 0.11, 0.33) were positively associated with ALM, whereas high‐density lipoprotein cholesterol (−0.53; −0.81, –0.25) was inversely associated with ALM.

**Conclusions:**

Sociodemographic, lifestyle, anthropometric, clinical, biochemical and treatment‐related factors show independent associations with longitudinal changes in ALM.

**Policy Impact**

This study provides long‐term evidence on determinants of muscle ageing, supporting policies that prioritise early prevention. Public health initiatives promoting exercise, nutrition and cardiovascular monitoring can mitigate sarcopenia's burden, reduce healthcare costs and improve functional independence in ageing populations.

## 1 Introduction

Sarcopenia is an age‐related condition characterised by progressive loss of skeletal muscle mass and function [1]. Contemporary working groups, including the European Working Group on Sarcopenia in Older People 2 (EWGSOP2), the Asian Working Group for Sarcopenia (AWGS) and the Foundation for the National Institutes of Health (FNIH), all include muscle mass, or its surrogate measure, lean mass, in their definitions [2, 3]. While there is no universally agreed upon operational definition, sarcopenia represents a major public health challenge, affecting approximately 10%–15% of the global ageing population, with prevalence rising to nearly 50% among individuals aged 80 years and older [2, 4]. Age‐related declines in muscle mass begin as early as mid‐adulthood and accelerate with advancing age, with men experiencing a steeper absolute loss due to higher peak muscle mass, and women showing an accelerated decline after menopause as oestrogen levels fall [5]. These trajectories underscore the importance of preserving healthy lean mass across the lifespan, as reduced lean mass is strongly linked to impaired mobility, balance deficits, glucose dysregulation, frailty, falls, fractures, disability and loss of independence [6].

Understanding the determinants of lean mass is essential for developing targeted interventions to maintain physical function and independence in ageing populations. Although multiple factors influence the preservation of lean mass, longitudinal evidence remains inconsistent and the relative contribution of lifestyle, nutrition, comorbid conditions and medication use is not fully established [7]. Chronic low‐grade inflammation, age‐related endocrine dysregulation and the accumulation of chronic conditions accelerate muscle catabolism through increased cytokine activity, reduced anabolic hormone availability and declines in physical activity. These interconnected processes impair muscle protein synthesis, promote muscle wasting and progressively weaken the musculoskeletal system [8].

Despite increasing research in sarcopenia, gaps in identifying modifiable determinants of preserving lean mass remain a critical area of investigation. Longitudinal cohort studies are uniquely designed to explore temporal relationships and inform preventive strategies. Therefore, this study aimed to investigate the time‐varying determinants of lean mass over an extended period, with a focus on lifestyle behaviours, anthropometry, body composition, blood biomarkers and comorbid conditions using 17 years of data from a population‐based cohort study of men and women.

## 2 Methods

### 2.1 Study Design and Participants

This longitudinal study utilised data from the Geelong Osteoporosis Study (GOS), a population‐based cohort study situated in southeastern Australia. Participants were randomly selected from the Australian electoral roll for the Barwon Statistical Division and included 1494 women (recruited 1993–1997) and 1539 men (recruited 2001–2006), aged 20–96 years. For the current study, data from multiple assessment waves were included: baseline, 10‐ and 15‐year follow‐up visits for women and baseline, 5‐ and 15‐year follow‐up visits for men.

Lifestyle and comorbidity data were obtained via self‐report at each visit. Anthropometry, body composition and clinical factors were measured at baseline and subsequent follow‐ups. Blood samples were collected after an overnight fast at baseline and 10‐year follow‐up for women and baseline and 15‐year follow‐up for men; multiple aliquots of serum and plasma were stored at −80°C until batch analyses. A full description of the GOS dataset has been published elsewhere [9]. This study was approved by the Barwon Health Human Research Ethics Committee (ID 92/01 and 00/56). All participants provided written informed consent.

### 2.2 Measures

#### 2.2.1 Exposures

Area‐based socio‐economic status (SES) was determined at baseline using the Index of Relative Socio‐economic Disadvantage (IRSD), as defined by the Australian Bureau of Statistics (ABS), based on socio‐economic indices for area codes corresponding to participant addresses. For this study, IRSD was categorised as low (1st and 2nd quintiles), medium (3rd quintile) and high SES (4th and 5th quintiles). The primary exposures in the analysis included smoking (current/past/never), frequency of alcohol consumption categorised as low (never/once in a week/twice a week) or high (several times a week/everyday), and physical activity based on mobility, grouped as active (very active and active) or inactive (sedentary, limited, bedfast and bedridden). Dietary calcium intake measured in milligrams per day was obtained using a validated questionnaire initially [10] and subsequently using the Dietary Questionnaire for Epidemiological Studies (DQES v3.2), from the Cancer Council Victoria, Australia [11] Dietary calcium intake was expressed in grams per day (g/day).

Body mass index (BMI) was calculated as weight in kilograms divided by the square of height in metres (kg/m^2^). Total fat mass was obtained from whole‐body dual‐energy X‐ray absorptiometry (DXA) and recorded in grams; values were converted to kilograms for analysis. Waist circumference (the smallest circumference between the lower rib and iliac crest) was measured in centimetres using a standardised protocol with a narrow, non‐stretch anthropometric tape. Bone mineral density (BMD), expressed in grams per square centimetre (g/cm^2^), was obtained at the lumbar spine and femoral neck by dual‐energy X‐ray absorptiometry (DXA) using a Lunar densitometer (GE Healthcare, Madison, WI, USA). *T*‐scores were calculated using sex‐specific Australian young‐adult reference data [12, 13]. A DPX‐L densitometer was used at baseline and 10‐year follow‐up in women and replaced by the Prodigy Pro at the 15‐year follow‐up. In men, the DPX‐L was used at baseline before transitioning to the Prodigy Pro for all subsequent scans. Cross‐calibration procedures were undertaken to maintain comparability between devices over time [9].

Biochemical markers were assessed from blood samples after an overnight fast. The analytes included fasting plasma glucose, serum procollagen type 1 N‐terminal propeptide (P1NP), C‐terminal telopeptide of type I collagen (CTx), high‐sensitivity C‐reactive protein (hsCRP), high‐density lipoprotein cholesterol (HDL‐C), low‐density lipoprotein cholesterol (LDL‐C), total cholesterol (TC) and triglycerides. All biomarkers were analysed as continuous variables except plasma glucose, which was categorised into two groups (low < 7 mmol/L and high ≥ 7 mmol/L) [14]. Bone turnover markers (CTx and P1NP) and hsCRP measured in mg/L were rescaled by dividing by 100 (unit: 0.01 mg/L) to improve numerical stability in the models.

Systolic and diastolic blood pressure (BP) were measured using a calibrated digital sphygmomanometer at each visit. Comorbidities were self‐reported via questionnaire. Participants were asked to indicate whether they had ever been diagnosed with any of the following conditions: cancer, asthma, emphysema, stroke, hypertension or osteoarthritis, with age of onset recorded. Falls in the prior 12 months were also reported. Medication use was documented by questionnaire and verified with containers or lists of medications brought to appointments; included were glucocorticoids, gonadal and thyroid hormones, and agents affecting calcium metabolism. Mortality data were obtained through linkage with the Australian Institute of Health and Welfare.

#### 2.2.2 Outcome Variable

Appendicular lean mass (ALM) was calculated as the sum of lean mass in the arms and legs, as measured by whole‐body DXA scans. For participants whose limbs extended beyond the width of the scanning field, the manufacturer's mirror‐mode function was used to estimate the missing segment by reflecting the contralateral limb, in accordance with standard DXA procedures. Where DXA mirror‐mode was used, data were checked for implausible values. Lean mass assessed by DXA represents all non‐fat and non‐bone tissues and aligns closely with skeletal muscle mass measured by magnetic resonance imaging (with correlations of 𝑟 = 0.94 for the whole body and 𝑟 = 0.91 for the leg) [15].

### 2.3 Statistical Analysis

Baseline and follow‐up characteristics were summarised as counts and percentages for categorical variables, means and standard deviations (SDs) for normally distributed continuous variables and medians with interquartile ranges (IQRs) for skewed continuous variables. Variables were selected based on prior evidence, biological plausibility and their established relevance to musculoskeletal ageing. Determinants of lean mass were analysed using linear mixed effect models with participant‐specific random intercepts, implemented in the *nlme* package in R 4.3.1 and fitted using restricted maximum likelihood (REML). Follow‐up time was modelled as a continuous variable. Random slopes for time were formally evaluated by fitting models with individual‐specific slopes in addition to random intercepts and comparing them using likelihood‐ratio tests. Although random‐slope models showed statistically significant improvements in the likelihood, they did not converge reliably or improve interpretability because a maximum of only three repeated measurements were available per participant. Accordingly, a random intercept structure was retained as the most parsimonious and stable specification. Repeatedly measured variables, including lifestyle behaviours, anthropometry, clinical conditions, biochemical markers and medication use, were treated as time‐varying covariates, while fixed characteristics such as sex and baseline age were modelled as time‐invariant. Sex–time and sex–exposure interactions were evaluated to assess potential effect modification; however, these terms did not improve model fit or contribute meaningfully to interpretation and were therefore not retained. Sex was included as a fixed effect in all models. Non‐linearity for continuous covariates (follow‐up duration, dietary calcium intake, BMI, fat mass, CTx, P1NP, hsCRP and systolic blood pressure) was assessed by introducing quadratic terms into the time varying mixed effects model. Quadratic terms were retained only when they materially improved model fit and were statistically significant. The significance and fitted shapes of these terms were used to evaluate departures from linearity, allowing the model to capture curvature in the associations between ALM and each covariate. Model‐derived curvature plots further illustrated the presence and direction of these nonlinear relationships.

To minimise multi‐collinearity, we examined correlation matrices for all covariates and avoided including highly correlated variables in the same model (e.g., BMI and fat mass). As a result, no pair of strongly correlated predictors was retained concurrently in the final models. In addition, variance inflation factors calculated from an equivalent fixed effects model indicated no concerning multicollinearity among the included predictors. No formal adjustment for multiple comparisons was applied because the analyses were based on pre‐specified predictors informed by prior literature and biological plausibility, rather than exploratory hypothesis testing.

Missing data in covariates were addressed using multiple imputation with predictive mean matching implemented in the *mice* package in R. A single imputed dataset (*m* = 1) was generated, and the imputation model included all variables used in the mixed effect analyses. Imputation was performed across all measurement waves to preserve the longitudinal structure of the data. Sensitivity analyses comparing imputed and complete‐case models produced highly consistent estimates, indicating that the imputation procedure did not materially influence the results. Statistical significance was set at *p* < 0.05.

To address survivorship bias, sensitivity analyses compared results from the full dataset with those restricted to participants completing at least one follow‐up. A second analysis replaced BMI with fat mass to test whether associations with cardiometabolic markers reflected general adiposity rather than weight‐for‐height. Robustness was further assessed using a complete‐case analysis without imputation, excluding participants with missing covariates as required by mixed‐effects models. Dietary calcium intake, body composition measures (including ALM), systolic BP and biochemical markers were modelled as continuous covariates, except plasma glucose, which was dichotomised. Treating predictors as continuous enabled assessment of linearity in their associations with ALM across follow‐up.

## 3 Results

A total of 3033 individuals (*n* = 1494 women, *n* = 1539 men; median age 55.3 years) were assessed at baseline. The median follow‐up duration was 16.7 years (IQR 9.7–23.2). At the first follow‐up for this analysis, 1859 participants (*n* = 881 women, *n* = 978 men; median age 58.6 years) returned. At the second follow‐up for this analysis, 1290 participants (*n* = 701 women, *n* = 589 men; median age 62.3 years) attended. Reasons for loss to follow‐up included death (*n* = 820), relocation out of the region (*n* = 103), inability to be contacted (*n* = 325), inability to provide consent (*n* = 10) and declined participation (*n* = 485).

The baseline characteristics of the cohort are summarised in Table 1. Socio‐economic status was represented across low, medium and high categories, and about half had never smoked. Approximately one‐third of participants reported high alcohol intake and nearly three‐quarters were physically active. Body composition and bone health measures reflected generally healthy anthropometric and densitometric profiles. Cardiometabolic markers, including blood pressure, lipids, glucose and inflammatory biomarkers, were broadly within expected ranges for a community‐dwelling population. Chronic conditions such as diabetes, osteoarthritis, cancer and respiratory diseases were relatively uncommon, and only a small proportion reported use of glucocorticoids, gonadal or thyroid hormones or calcium‐affecting agents.

**TABLE 1:** Characteristics of the cohort at baseline.

Characteristics	Baseline (*n* = 3033)
Age (years)	55.3 (38.4, 72.5)
Female sex	1494 (49%)
SES	
Low (referent)	1196 (39%)
Medium	604 (20%)
High	1233 (41%)
Smoking	
Never smoked (referent)	1534 (51%)
Past smokers	1026 (34%)
Current smokers	473 (16%)
High alcohol intake	967 (32%)
Physically active	2141 (71%)
Dietary calcium intake (g/day)	0.8 (0.5, 1.0)
Systolic BP (mmHg)	130.0 (20.5)
Diastolic BP (mmHg)	80.3 (13.0)
Waist circumference (cm)	91.2 (13.7)
ALM (kg)	21.6 (5.7)
Fat mass (kg)	23.9 (10.0)
Weight (kg)	75.4 (16.2)
Height (cm)	167.5 (10.1)
BMI (kg/m^2^)	26.2 (23.5, 29.3)
ALM/h^2^ (kg/m^2^)	7.5 (6.5, 8.6)
Femoral neck *T*‐score	−0.9 (1.2)
Spine *T*‐score	−0.3 (1.5)
P1NP (μg/L)	37.0 (27.0, 50.0)
CTx (ng/L)	334.0 (226.5, 474.0)
hsCRP (mg/L)	3.9 (7.4)
Total cholesterol (mmol/L)	5.3 (1.1)
LDL cholesterol (mmol/L)	3.1 (0.9)
HDL cholesterol (mmol/L)	1.3 (0.3)
Triglycerides (mmol/L)	1.5 (0.9)
High plasma glucose (≥ 7 mmol/L)	116 (4%)
Falls in the past year	677 (22%)
Diabetes	192 (6%)
Osteoarthritis	323 (11%)
Cancer	338 (11%)
Stroke	96 (3%)
Cardiac arrhythmias	77 (3%)
Transient weakness	44 (2%)
Asthma	407 (13%)
Emphysema	55 (2%)
Bronchitis	88 (3%)
Oral glucocorticoids	57 (2%)
Gonadal hormones	173 (6%)
Thyroid hormones	50 (2%)
Agent affecting calcium	42 (1%)

*Note:* Values are presented as mean (SD), median (Q1–Q3) or *n* (%). Medications included in ‘agents affecting calcium’: Vitamin D supplements, bisphosphonates (including risedronate, alendronate, etidronate and denosumab), selective estrogen reuptake inhibitors, strontium ranelate. Medications included in ‘gonadal hormones’: Oestrogen therapies (including Premarin, Ogen, Progynova), progesterone/progestins (including Provera, Primolut N, Depo‐Provera) and occasional androgen preparations (including testosterone implants, Androderm). Medications included ‘thyroid hormones’: Levothyroxine preparations (including Thyroxine, Oroxine, Eutroxsig) prescribed for hypothyroidism or post‐thyroidectomy replacement, and antithyroid agents used for hyperthyroidism (including Carbimazole/Neo‐Mercazole, Propylthiouracil).

Abbreviations: ALM, appendicular lean mass; BMI, body mass index; BP, blood pressure; CRP, C‐reactive protein; CTx, C‐terminal telopeptide of type I collagen; HDL, high‐density lipoprotein; LDL, low‐density lipoprotein; P1NP, procollagen type 1 N‐terminal propeptide; SES, socio‐economic status.

### 3.1 Linear Mixed Effect Model

In the BMI‐adjusted mixed effects model (Table 2), ALM declined over time, with each additional year of follow‐up associated with a 0.16 kg lower ALM, and the small positive quadratic term (0.01) indicating a modest deceleration in decline as follow‐up progressed. Older age was associated with lower ALM, with each additional year of age corresponding to a 0.07 kg lower ALM. Women had substantially lower ALM than men (−8.36 kg). Higher SES was associated with greater ALM, with medium and high SES corresponding to 0.19 kg and 0.33 kg higher ALM, respectively, compared with low SES. Higher dietary calcium intake was associated with greater ALM, with each additional gram per day corresponding to a 0.46 kg higher ALM. Higher femoral neck T‐scores were also associated with greater ALM, with each one unit increase in T‐score corresponding to a 0.38 kg higher ALM. Smoking and physical inactivity were each associated with lower ALM, with past smokers having 0.24 kg lower ALM and physically inactive participants having 0.48 kg lower ALM. Body Mass Index demonstrated a strong non‐linear association with ALM: each 1 kg/m² increase in BMI was associated with a 0.55 kg higher ALM, but the negative quadratic term indicates that this increase slowed and eventually plateaued at very high BMI values. Inflammatory markers showed a pronounced U‐shaped relationship, with lower ALM at both low and high hsCRP levels: each 0.01 mg/L increase in hsCRP was associated with a 10.38 kg lower ALM at the lower values, while the positive quadratic term (11.70) indicates rising ALM at higher hsCRP values. C‐terminal telopeptide (CTx) also demonstrated a non‐linear association with ALM: each 1 ng/L increase in CTx was associated with a 0.14 kg higher ALM at lower values, but the negative quadratic term (−0.01) indicates progressively lower predicted ALM at higher CTx values. Systolic BP showed a significant positive association with ALM, with each 1 mmHg increase associated with a 0.06 kg higher ALM, and the quadratic term indicating a slight inverted U‐shaped pattern. In addition, stroke was associated with 0.51 kg lower ALM, while thyroid hormone therapy was associated with 0.52 kg higher ALM.

**TABLE 2:** Multivariable mixed effects model examining determinants of appendicular lean mass over 16 years of follow‐up (BMI‐adjusted model).

Predictors	Estimates	95% CI	*p*
(Intercept)	13.20	10.56 to 15.85	**< 0.001**
Follow‐up (years)	−0.16	−0.20 to −0.13	**< 0.001**
Follow‐up^2^ (years)	0.01	0.00 to 0.01	**< 0.001**
Age at baseline (years)	−0.07	−0.08 to −0.06	**< 0.001**
Female sex	−8.36	−8.58 to −8.15	**< 0.001**
Past smokers	−0.24	−0.46 to −0.03	**0.03**
Current smokers	−0.21	−0.48 to 0.06	0.12
Medium SES	0.19	−0.01 to 0.40	0.06
High SES	0.33	0.14 to 0.51	**0.001**
Low alcohol intake	−0.11	−0.30 to 0.08	0.27
Physical Inactivity	−0.48	−0.65 to −0.31	**< 0.001**
Dietary calcium intake (g/day)	0.46	0.25 to 0.67	**< 0.001**
LDL cholesterol (mmol/L)	0.03	−0.06 to 0.13	0.47
HDL cholesterol (mmol/L)	−0.08	−0.35 to 0.18	0.53
Triglycerides (mmol/L)	0.07	−0.03 to 0.18	0.17
BMI (kg/m^2^)	0.55	0.45 to 0.66	**< 0.001**
BMI^2^ (kg/m^2^)	−0.00	−0.01 to −0.00	**< 0.001**
hsCRP (0.01 mg/L)	−10.38	−12.66 to −8.10	**< 0.001**
hsCRP^2^ (0.01 mg/L)	11.70	8.30 to 15.10	**< 0.001**
CTx (ng/L)	0.14	0.07 to 0.21	**< 0.001**
CTx^2^ (ng/L)	−0.01	−0.01 to −0.00	**0.03**
Femoral neck *T*‐score	0.38	0.29 to 0.47	**< 0.001**
Systolic BP (mmHg)	0.06	0.03 to 0.10	**< 0.001**
Systolic BP^2^ (mmHg)	−0.00	‐0.00 to −0.00	**< 0.001**
High glucose (≥ 7 mmol/L)	−0.21	−0.59 to 0.17	0.28
Falls	−0.14	−0.31 to 0.02	0.08
Stroke	−0.51	−0.96 to −0.07	**0.02**
Cardiac arrhythmias	0.21	−0.16 to 0.59	0.27
Asthma	−0.13	−0.36 to 0.10	0.27
Emphysema	−0.14	−0.72 to 0.45	0.65
Bronchitis	0.20	−0.22 to 0.62	0.35
Cancer	−0.01	−0.23 to 0.20	0.91
Osteoarthritis	0.01	−0.21 to 0.23	0.93
Oral glucocorticoids	0.02	−0.49 to 0.53	0.93
Gonadal hormones	0.27	−0.07 to 0.61	0.12
Thyroid hormones	0.52	0.04 to 0.99	**0.03**
Agent affecting calcium	0.02	−0.47 to 0.51	0.93
**Random effects**			
*σ* ^2^	4.60		
*τ*_00 id_	4.70		
ICC	0.51		
*N*_id_	3033		
Marginal *R* ^2^/conditional *R* ^2^	0.708/0.855		

*Note:* Values are fixed effect estimates with 95% confidence intervals from a linear mixed effects model with random intercepts for participants. The outcome is appendicular lean mass (kg). Follow‐up time, BMI, systolic blood pressure, hsCRP and CTx were modelled with linear and quadratic terms. Reference categories: sex = male; smoking = never smoker; socio‐economic status = low SES; alcohol intake = high; physical activity = active; glucose = < 7 mmol/L; no history of falls, stroke, cardiac arrhythmia, asthma, emphysema, bronchitis, cancer, osteoarthritis; and non‐use of oral glucocorticoids, gonadal hormones, thyroid hormones or calcium‐affecting agents. *R* ^2^ values represent marginal (fixed effects only) and conditional (fixed + random effects) variance explained.

Abbreviations: ALM, appendicular lean mass; BMD, bone mineral density; BMI, body mass index; BP, blood pressure; CTx, C‐terminal telopeptide of type I collagen; HDL, high‐density lipoprotein; hsCRP, high‐sensitivity C‐reactive protein; ICC, intra‐class correlation coefficient LDL, low‐density lipoprotein; SES, socio‐economic status.

In the sensitivity analysis replacing BMI with DXA‐derived fat mass (Table 3), the overall pattern of associations remained consistent with the primary model. However, fat mass demonstrated a strong non‐linear association with ALM, with the highest ALM at lower fat mass, but with a progressive decline as fat mass increased; each 1 kg increase in fat mass was associated with a 0.11 kg higher ALM at lower levels, with the negative quadratic term indicating declining ALM at higher fat mass values. Cardiometabolic markers showed clearer associations in this model: higher LDL and triglycerides were associated with greater ALM with each 1 mmol/L increase corresponding to 0.14 kg and 0.22 kg higher ALM, respectively, whereas HDL was inversely associated, with each 1 mmol/L increase corresponding to a 0.53 kg lower ALM. Systolic BP showed a stronger association than in the BMI‐adjusted model, with each 1 mmHg increase associated with a 0.12 kg higher ALM.

**TABLE 3:** Multivariable mixed effects model examining determinants of appendicular lean mass over 16 years of follow‐up (fat mass‐adjusted model).

Predictors	Estimates	95% CI	*p*
(Intercept)	19.09	16.71 to 21.47	**< 0.001**
Follow‐up (years)	−0.13	−0.17 to −0.09	**< 0.001**
Follow‐up^2^ (years)	0.01	0.00 to 0.01	**< 0.001**
Age at baseline (years)	−0.07	−0.08 to −0.06	**< 0.001**
Female sex	−8.42	−8.66 to −8.19	**< 0.001**
Past smokers	−0.08	−0.31 to 0.16	0.52
Current smokers	−0.35	−0.64 to −0.06	**0.02**
Medium SES	0.12	−0.10 to 0.33	0.29
High SES	0.26	0.06 to 0.45	**0.01**
Low alcohol intake	−0.07	−0.27 to 0.13	0.49
Physical Inactivity	−0.29	−0.46 to −0.11	**0.001**
Dietary calcium intake (g/day)	0.42	0.20 to 0.64	**< 0.001**
LDL cholesterol (mmol/L)	0.14	0.04 to 0.24	**0.007**
HDL cholesterol (mmol/L)	−0.53	−0.81 to −0.25	**< 0.001**
Triglycerides (mmol/L)	0.22	0.11 to 0.33	**< 0.001**
Fat mass (kg)	0.11	0.07 to 0.14	**< 0.001**
Fat mass^2^ (kg)	−0.00	−0.00 to −0.00	**< 0.001**
hsCRP (0.01 mg/L)	−6.72	−9.13 to −4.31	**< 0.001**
hsCRP^2^ (0.01 mg/L)	8.24	4.63 to 11.86	**< 0.001**
CTx (ng/L)	0.11	0.03 to 0.18	**0.008**
CTx^2^ (ng/L)	−0.01	−0.01 to −0.00	**0.04**
Femoral neck *T*‐score	0.63	0.54 to 0.72	**< 0.001**
Systolic BP (mmHg)	0.12	0.09 to 0.15	**< 0.001**
Systolic BP^2^ (mmHg)	−0.00	‐0.00 to −0.00	**< 0.001**
High glucose (≥ 7 mmol/L)	0.01	−0.40 to 0.41	0.97
Falls	−0.10	−0.27 to 0.08	0.28
Stroke	−0.50	−0.97 to −0.03	**0.04**
Cardiac arrhythmias	0.12	−0.28 to 0.51	0.56
Asthma	0.03	−0.21 to 0.28	0.78
Emphysema	−0.24	−0.85 to 0.38	0.45
Bronchitis	0.25	−0.19 to 0.69	0.27
Cancer	−0.08	−0.31 to 0.15	0.497
Osteoarthritis	0.05	−0.17 to 0.28	0.64
Oral glucocorticoids	−0.05	−0.58 to 0.49	0.87
Gonadal hormones	0.09	−0.26 to 0.45	0.61
Thyroid hormones	0.75	0.26 to 1.25	**0.003**
Agent affecting calcium	−0.15	−0.67 to 0.36	0.56
**Random effects**			
*σ* ^2^	4.92		
*τ*_00 id_	5.76		
ICC	0.54		
*N*_id_	3033		
Marginal *R* ^2^/conditional *R* ^2^	0.665/0.845		

*Note:* Values are fixed effect estimates with 95% confidence intervals from a linear mixed effects model with random intercepts for participants. The outcome is appendicular lean mass (kg). Follow‐up time, fat mass, systolic blood pressure, hsCRP and CTx were modelled with linear and quadratic terms. Reference categories: sex = male; smoking = never smoker; socio‐economic status = low SES; alcohol intake = high; physical activity = active; glucose = < 7 mmol/L; no history of falls, stroke, cardiac arrhythmia, asthma, emphysema, bronchitis, cancer, osteoarthritis; and non‐use of oral glucocorticoids, gonadal hormones, thyroid hormones, or calcium‐affecting agents. *R* ^2^ values represent marginal (fixed effects only) and conditional (fixed + random effects) variance explained.

Abbreviations: ALM, appendicular lean mass; BMD, bone mineral density; BP, blood pressure; CTx, C‐terminal telopeptide of type I collagen; HDL, high‐density lipoprotein; hsCRP, high‐sensitivity C‐reactive protein; ICC, intra‐class correlation coefficient; LDL, low‐density lipoprotein; SES, socio‐economic status.

Model fit was compared between a linear time model and a quadratic time model using the Akaike information criterion (AIC). The quadratic model showed improved fit (AIC = 30,451.80) compared with the linear model (AIC = 30,519.87), yielding a ΔAIC of 68.07. This supports the inclusion of a non‐linear time term in the model.

Visualisation of the quadratic follow‐up term showed a steady decline in ALM over most of the period, with a slight rise after ~15 years (Figure 1a), likely indicating a survivorship pattern. Body mass index displayed a non‐linear pattern, increasing at lower and mid‐range values and plateauing at higher levels (Figure 1b). Systolic blood pressure showed an inverted U‐shape, with peak ALM around 140 mmHg (Figure 1c). Serum hsCRP demonstrated a U‐shaped association (Figure 1d), whereas CTx showed a largely monotonic decline in ALM with increasing levels (Figure 1e). Fat mass exhibited a similar pattern, with ALM highest at lower fat mass and progressively decreasing with greater adiposity (Figure 1f).

**FIGURE 1:**
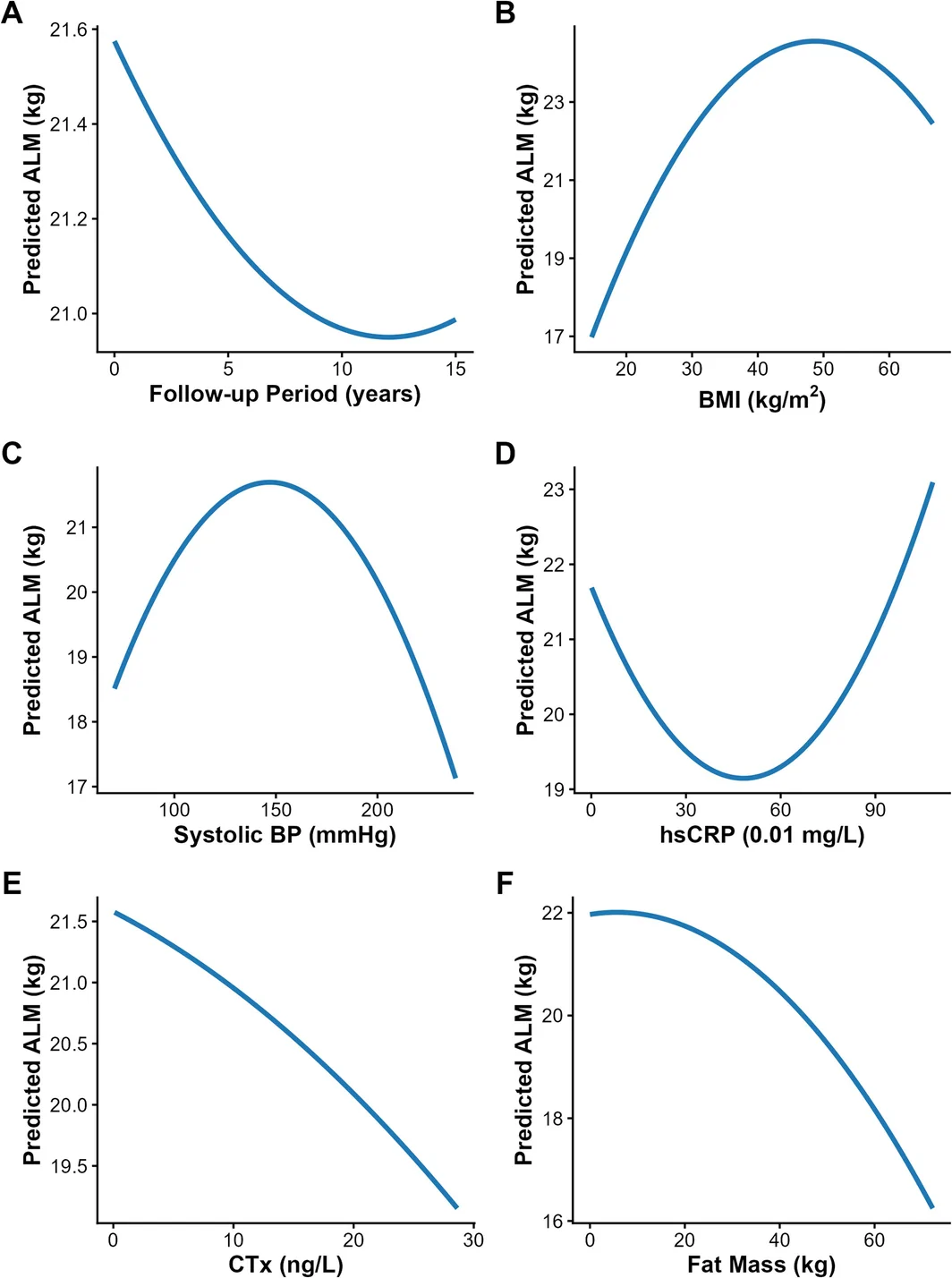
Model‐derived curvature plots showing nonlinear associations between appendicular lean mass and selected predictors. Panels depict predicted ALM from the multivariable mixed effects model, including quadratic terms for (a), follow‐up time (b), body mass index (c), systolic blood pressure (d), high‐sensitivity C‐reactive protein (e), serum C‐terminal telopeptide of type I collagen and (f), fat mass. ALM, appendicular lean mass; BMI, body mass index; BP, blood pressure; CTx, C‐terminal telopeptide of type I collagen; Fu, follow‐up time, hsCRP, high‐sensitivity C‐reactive protein.

Another sensitivity analysis restricted to participants with at least one follow‐up (Table 4) yielded results highly consistent with the main model. The direction and significance of key associations remained unchanged, with only slight attenuation of effect sizes, reflecting the healthier subset who returned for follow‐up. Overall, selective follow‐up did not materially influence the findings. Similarly, the complete‐case analysis produced effect estimates comparable to those from the imputed dataset, with no substantive differences observed in the significance or interpretation of the predictors.

**TABLE 4:** Multivariable mixed effects model examining determinants of appendicular lean mass over 16 years of follow‐up (sensitivity analysis of participants with ≥ 2 follow‐up [*n* = 1920]).

Predictors	Estimates	95% CI	*p*
(Intercept)	14.29	11.36 to 17.23	**< 0.001**
Follow‐up (years)	−0.17	−0.21 to −0.13	**< 0.001**
Follow‐up^2^ (years)	0.01	0.00 to 0.01	**< 0.001**
Age at baseline (years)	−0.07	−0.08 to −0.06	**< 0.001**
Female sex	−8.75	−9.01 to −8.49	**< 0.001**
Past smokers	−0.33	−0.58 to −0.07	**0.01**
Current smokers	−0.25	−0.56 to 0.07	0.12
Medium SES	0.18	−0.04 to 0.41	0.11
High SES	0.30	0.09 to 0.50	**0.005**
Low alcohol intake	−0.08	−0.29 to 0.14	0.48
Physical Inactivity	−0.49	−0.68 to −0.31	**< 0.001**
Dietary calcium intake (g/day)	0.42	0.19 to 0.65	**< 0.001**
LDL cholesterol (mmol/L)	0.10	−0.01 to 0.20	0.08
HDL cholesterol (mmol/L)	−0.03	−0.33 to 0.27	0.84
Triglycerides (mmol/L)	0.06	−0.06 to 0.18	0.33
BMI (kg/m^2^)	0.56	0.44 to 0.67	**< 0.001**
BMI^2^ (kg/m^2^)	−0.00	−0.01 to −0.00	**< 0.001**
hsCRP (0.01 mg/L)	−9.74	−12.28 to −7.20	**< 0.001**
hsCRP^2^ (0.01 mg/L)	10.75	6.96 to 14.54	**< 0.001**
CTx (ng/L)	0.15	0.06 to 0.23	**0.001**
CTx^2^ (ng/L)	−0.01	−0.01 to −0.00	**0.02**
Femoral neck *T*‐score	0.39	0.29 to 0.49	**< 0.001**
Systolic BP (mmHg)	0.05	0.02 to 0.09	**0.005**
Systolic BP^2^ (mmHg)	−0.00	‐0.00 to −0.00	**0.009**
High glucose (≥ 7 mmol/L)	−0.20	−0.62 to 0.23	0.37
Falls	−0.17	−0.35 to 0.00	0.05
Stroke	−0.41	−0.94 to 0.13	0.13
Cardiac arrhythmias	0.11	−0.29 to 0.50	0.61
Asthma	−0.09	−0.35 to 0.17	0.51
Emphysema	−0.20	−0.91 to 0.51	0.58
Bronchitis	0.38	−0.07 to 0.83	0.10
Cancer	0.01	−0.23 to 0.25	0.94
Osteoarthritis	0.01	−0.22 to 0.25	0.92
Oral glucocorticoids	0.03	−0.55 to 0.61	0.91
Gonadal hormones	0.25	−0.11 to 0.62	0.17
Thyroid hormones	0.49	−0.03 to 1.01	0.06
Agent affecting calcium	0.06	−0.47 to 0.60	0.82
**Random effects**			
*σ* ^2^	4.61		
*τ*_00 id_	4.74		
ICC	0.51		
*N*_id_	1920		
Marginal *R* ^2^/conditional *R* ^2^	0.707/0.855		

*Note:* Values are fixed effect estimates with 95% confidence intervals from a linear mixed effects model with random intercepts for participants. The outcome is appendicular lean mass (kg). Follow‐up time, BMI, systolic blood pressure, hsCRP and CTx were modelled with linear and quadratic terms. Reference categories: sex = male; smoking = never smoker; socio‐economic status = low SES; alcohol intake = high; physical activity = active; glucose = < 7 mmol/L; no history of falls, stroke, cardiac arrhythmia, asthma, emphysema, bronchitis, cancer, osteoarthritis; and non‐use of oral glucocorticoids, gonadal hormones, thyroid hormones or calcium‐affecting agents. *R* ^2^ values represent marginal (fixed effects only) and conditional (fixed + random effects) variance explained.

Abbreviations: ALM, appendicular lean mass; BMD, bone mineral density; BMI, body mass index; BP, blood pressure; CTx, C‐terminal telopeptide of type I collagen; HDL, high‐density lipoprotein; hsCRP, high‐sensitivity C‐reactive protein; ICC, intra‐class correlation coefficient; LDL, low‐density lipoprotein; SES, socio‐economic status.

## 4 Discussion

This study investigated determinants of ALM in a large Australian cohort, identifying sociodemographic, biological and lifestyle factors influencing lean mass over time. Although contemporary definitions of sarcopenia emphasise muscle strength as the primary indicator, with muscle mass used as a confirmatory measure, our study focused specifically on ALM because measures of muscle strength and physical performance were not available at the early phases of the study. Appendicular Lean Mass provides an objective estimate of lean mass quantity and is widely used in epidemiological studies of musculoskeletal ageing; however, it does not capture muscle strength, function or quality. Therefore, ALM does not reflect sarcopenia as defined by recent consensus frameworks, including the Global Leadership Initiative on Sarcopenia (GLIS), which recommends muscle strength as the primary criterion, lean mass as confirmatory and physical performance for severity grading [16]. Our findings should thus be interpreted as associations with muscle mass rather than with sarcopenia status.

The high conditional R², substantial Intraclass Correlation Coefficient, and stable random effects estimates observed across all models indicate that the associations identified in the mixed effects models are robust and consistent. Sensitivity analyses restricted to participants with at least one follow‐up produced consistent results, suggesting that selective follow‐up did not materially bias the estimated associations. However, the modest increase in ALM observed at the longest follow‐up is unlikely to represent a true biological improvement in lean mass and is more likely attributable to survivorship bias. Participants who remained in the cohort at later waves were substantially younger, healthier, and more physically active at baseline. This selective retention of healthier individuals likely leads to an over‐representation of participants with higher ALM at later time points, creating the appearance of late improvement despite the expected age‐related decline.

Replacing BMI with DXA‐derived fat mass yielded similar overall patterns of association, but fat mass itself demostrated a strong nonlinear relationship with ALM, showing highest ALM at lower fat mass and a progressive decline as fat mass increased [17]. In this model, LDL, HDL and triglycerides showed stronger associations with ALM than in the BMI‐adjusted model. This suggests that part of the cardiometabolic signal is mediated through adiposity, and that fat mass provides a more specific adjustment than BMI. Importantly, the direction of associations remained consistent across models, supporting the robustness of the findings.

### 4.1 Socio‐Demographics and Lifestyle Factors

Age at baseline and sex were strongly associated with ALM, with older age and female sex linked to lower ALM over time, consistent with the well‐described age and sex‐related patterns in sarcopenia epidemiology [18] Follow‐up duration was also linked to lower ALM, though a significant quadratic term reflected a non‐linear association. The observed pattern indicated a progressive decline with modest late‐phase attenuation, suggesting that muscle loss is not strictly linear and may reflect a combination of physiological adaptation, changes in exposure to risk factors, clinical interventions and survivorship effects, which are often not captured in cross‐sectional or short‐term studies.

The association between SES and ALM aligned with previous reports linking higher SES to better musculoskeletal health [19, 20] but our larger sample and repeated measures strengthen the inference that these differences are sustained over time rather than reflecting a single time‐point disparity.

Regarding lifestyle behaviours, smoking and physical inactivity were associated with lower ALM, while higher dietary calcium intake was associated with greater ALM, reinforcing the established role of behavioural and nutritional factors in maintaining lean mass [21, 22] Modelling these factors longitudinally allowed us to capture within‐person changes in behaviour and their cumulative impact on muscle trajectories, an advantage over cross‐sectional studies that cannot disentangle age effects from cohort differences.

### 4.2 Metabolic and Clinical Factors

Body Mass Index showed a strong positive association with ALM, although the negative quadratic term indicated diminishing gains at higher BMI levels. This pattern highlighted higher ALM at moderate BMI and a slight decline at extreme BMI values, is consistent with previous reports [23] and align with the description of sarcopenic obesity, a phenotype characterised by excess adiposity and disproportionately low muscle mass [24]. Individuals with higher BMI may appear to have had greater ALM on average, yet the attenuated or declining ALM at the upper BMI range likely reflects the metabolic and inflammatory consequences of obesity, including insulin resistance, chronic low‐grade inflammation and impaired muscle protein synthesis [25] These findings demonstrate that the relationship between adiposity and lean mass is dynamic, non‐linear and sensitive to shifts in body composition over time.

Even after replacing BMI with DXA‐derived fat mass, the association between fat mass and ALM remained non‐linear. However, the curvature plot reflected the strength of the association rather than meaningful curvature; the smooth function was essentially linear, showing a steady decline in ALM as fat mass increased. This pattern aligns with a substantial body of literature demonstrating that excess adiposity is linked to reduced muscle mass, poorer muscle quality and impaired metabolic function [26]. However, most previous studies have relied on BMI as a proxy for adiposity, which limited their ability to distinguish the independent contributions of fat and lean compartments. By incorporating DXA‐derived fat mass, our analysis provided stronger evidence that increasing adiposity is associated with lower lean mass over time, independent of age, lifestyle factors and cardiometabolic markers. These findings contribute to the broader understanding of sarcopenic obesity [27]. However, it is important to note that ALM reflects the quantity of lean tissue rather than muscle strength or function. Individuals with higher BMI or greater fat mass may therefore exhibit higher lean mass due to larger body size, without corresponding improvements in muscle quality or functional capacity.

### 4.3 Bone Turnover

The bone turnover markers, CTx and P1NP, indicative of bone resorption and formation, respectively, were positively associated with ALM in preliminary models, although only CTx was retained in the final model due to collinearity. C‐terminal telopeptide (CTx) demonstrated an inverse association with ALM, with higher bone resorption consistently linked to lower ALM over time. This pattern suggests that lower bone turnover may be favourable for maintaining muscle mass, whereas excessive turnover may be detrimental, consistent with the concept of coordinated bone‐muscle crosstalk mediated through shared endocrine and paracrine pathways, including osteokines and myokines [28]. The curvature plot reinforced this interpretation, which is aligned with previous clinical studies reporting associations between CTx and muscle outcomes, including work demonstrating that higher CTx levels were linked to poorer lower‐limb muscle function in older adults at high risk of falls and fractures [29].

### 4.4 Inflammation

Serum hsCRP showed a significant nonlinear association with ALM, forming a U‐shaped curve: lean mass was lowest at moderate hsCRP levels and higher at both low and high concentrations. This suggests a more complex relationship than a simple linear decline. Prior studies have described distinct inflammatory phenotypes with varying effects on muscle atrophy [28, 30] but most relied on cross‐sectional designs, limiting their ability to capture dynamic inflammatory states. Our findings extended this by showing that the relationship between hsCRP and ALM varied across the distribution, likely reflecting different inflammatory states. Low hsCRP likely represents physiological health, whereas moderate elevations may indicate chronic low‐grade inflammation, often linked to adiposity, metabolic dysfunction and catabolic signalling [31]. Very high hsCRP levels may reflect acute inflammatory responses, which can transiently elevate hsCRP without necessarily indicating sustained catabolic activity [32]. As only 13% of participants had hsCRP > 10 mg/L, the U‐shaped pattern was unlikely to be driven by acute inflammation. Instead, it appears to reflect variation in chronic low‐grade inflammation, metabolic health and adiposity‐related body composition.

### 4.5 Lipid Biomarkers

Higher LDL and triglycerides were associated with greater ALM, whereas HDL showed an inverse association in the fat‐mass‐adjusted model. Although these findings may initially appear counterintuitive given the established cardiometabolic risks associated with dyslipidaemia, recent studies have reported that higher lipid concentrations often co‐occur with greater overall body mass, reflecting parallel increases in both fat and lean compartments [33]. In this context, the positive associations observed for LDL and triglycerides likely reflect the metabolic profile of individuals with higher adiposity who also maintain relatively greater lean mass. This interpretation aligns with emerging evidence describing metabolically distinct obesity phenotypes, including forms of sarcopenic obesity in which excess adiposity coexists with preserved or even elevated lean mass but reduced muscle quality [34]. It is equally important to acknowledge that the associations between lipid profile and lean mass should be interpreted in the context of potential statin use. Statin therapy lowers LDL cholesterol and triglycerides and has been associated with muscle‐related adverse effects in previous studies [35, 36]. It is therefore possible that statin use may have partially influenced the observed lipid‐lean mass associations. Moreover, ALM captures lean mass rather than muscle strength or function; higher lipid levels may be associated with greater lean mass without necessarily indicating better muscle quality or performance. This distinction may help contextualise the observed lipid–ALM associations.

### 4.6 Medication Use

The positive association between thyroid hormone therapy and ALM should be interpreted within the broader context of thyroid and muscle physiology. Previous studies have reported that restoration of euthyroid status through appropriate hormone therapy can reverse deficits in mitochondrial efficiency, muscle fibre composition and neuromuscular function [37, 38] Our findings were consistent with this evidence, suggesting that treated hypothyroidism and hyperthyroidism may be associated with better preservation of lean mass. However, because we did not measure thyroid hormone levels, treatment adequacy or TSH suppression, the underlying mechanisms remain uncertain, and reverse causation or differences in health‐seeking behaviour may also contribute.

### 4.7 Comorbid Conditions

Systolic BP showed a non‐linear association with ALM, forming an inverted U‐shaped pattern: lean mass was highest at mid‐range BP, while both lower and higher pressures were linked to reduced ALM. Similar findings have been reported [39, 40] though mechanisms remain unclear. Elevated BP may reflect vascular stiffness, endothelial dysfunction or metabolic dysregulation, whereas low BP may signal frailty or illness [39].

Stroke was negatively associated with ALM, consistent with the musculoskeletal consequences of cerebrovascular injury [41]. Muscle loss after stroke arises from motor impairment, reduced mobility, inflammation and neurogenic wasting, leading to rapid atrophy and long‐term risk of sarcopenia [42, 43]. Our findings aligned with this evidence and underscored the substantial musculoskeletal burden of stroke and its relevance to long‐term muscle health.

### 4.8 Strengths and Limitations

This study has several strengths. To our knowledge, it is one of the first long‐term population‐based studies to have examined determinants of ALM over nearly two decades using repeated DXA‐derived measurements. The large sample and high number of observations provided strong statistical power for the precise estimation of linear and nonlinear associations. Time‐varying mixed‐effects modelling accounted for within‐individual correlation and between‐individual variability, offering a rigorous approach to longitudinal analysis. Adjustment for a wide range of determinants reduced residual confounding, providing a more integrated view of factors associated with lean mass. Importantly, modelling nonlinear relationships captured complex physiological patterns often missed by linear models. The population‐based, randomly selected cohort and objectively measured exposures and outcomes further minimised selection and measurement bias.

Limitations should also be noted. Selective attrition and survivorship may have affected late‐phase trajectories; although sensitivity analyses showed consistent results, some bias cannot be excluded. Residual confounding remains possible; for example, detailed statin exposure data were not included in the primary models, and residual confounding by statin therapy cannot be excluded given its known effects on lipid levels and potential muscle‐related outcomes. Self‐reported variables may have introduced recall or reporting bias. The cohort's specific population may limit generalisability to other demographic or ethnic groups. Unmeasured factors such as dietary protein intake, detailed physical activity intensity, and hormonal profiles may also have influenced muscle mass but were not assessed. The DXA‐derived fat mass does not distinguish visceral, subcutaneous, and intra‐muscular fat, each with distinct effects on muscle health. Finally, while polynomial terms identified non‐linear patterns, more flexible approaches (e.g., splines) could further refine these associations in future work.

## 5 Conclusions

This study provides a comprehensive analysis of determinants of ALM, suggesting complex and non‐linear associations across physiological, biochemical and lifestyle domains. The non‐linear relationships observed for follow‐up duration, BMI, fat mass, CTx, hsCRP and systolic BP highlight interconnected influences of body composition, bone turnover, systemic inflammation and cardiometabolic health on lean mass over time. These results emphasise the value of integrated assessments that capture these multidimensional relationships, enabling a more complete understanding of the factors associated with muscle health and providing a basis for future work examining how these factors relate to muscle ageing across adulthood. Overall, the findings reinforce the importance of longitudinal, multidimensional approaches to understanding muscle health and support the use of curvature modelling to capture nonlinear dynamics.

## Funding

The Geelong Osteoporosis Study was funded by the National Health and Medical Research Council (NHMRC) of Australia with project numbers (projects 251638, 299831, 628582). Aminu Suleiman was supported by a Deakin University postgraduate research scholarship (DUPRS). KLH‐K was supported by an Alfred Deakin Postdoctoral Research Fellowship. LJW was supported by an NHMRC Emerging Leader Fellowship (ID 1174060).

## Ethics Statement

This study was conducted in accordance with the ethical principles of the Declaration of Helsinki. It followed Barwon Health human research ethical standards and was approved by the Barwon Health Human Research Ethics Committee (ID 92/01 and 00/56).

## Conflicts of Interest

The authors declare no conflicts of interest.

## Data Availability

The data that support the findings of this study are available on request from the corresponding author. The data are not publicly available due to privacy or ethical restrictions.
